# Promoting Social Distancing and COVID-19 Vaccine Intentions to Mothers: Randomized Comparison of Information Sources in Social Media Messages

**DOI:** 10.2196/36210

**Published:** 2022-08-23

**Authors:** David Buller, Barbara Walkosz, Kimberly Henry, W Gill Woodall, Sherry Pagoto, Julia Berteletti, Alishia Kinsey, Joseph Divito, Katie Baker, Joel Hillhouse

**Affiliations:** 1 Klein Buendel, Inc Golden, CO United States; 2 Department of Psychology Colorado State University Fort Collins, CO United States; 3 Department of Allied Health Sciences University of Connecticut Storrs, CT United States; 4 Department of Community and Behavioral Health East Tennessee State University Johnson City, TN United States

**Keywords:** social media, COVID-19, vaccination, nonpharmaceutical interventions, information source, misinformation, vaccine, public health, COVID-19 prevention, health promotion

## Abstract

**Background:**

Social media disseminated information and spread misinformation during the COVID-19 pandemic that affected prevention measures, including social distancing and vaccine acceptance.

**Objective:**

In this study, we aimed to test the effect of a series of social media posts promoting COVID-19 nonpharmaceutical interventions (NPIs) and vaccine intentions and compare effects among 3 common types of information sources: government agency, near-peer parents, and news media.

**Methods:**

A sample of mothers of teen daughters (N=303) recruited from a prior trial were enrolled in a 3 (information source) × 4 (assessment period) randomized factorial trial from January to March 2021 to evaluate the effects of information sources in a social media campaign addressing NPIs (ie, social distancing), COVID-19 vaccinations, media literacy, and mother–daughter communication about COVID-19. Mothers received 1 social media post per day in 3 randomly assigned Facebook private groups, Monday-Friday, covering all 4 topics each week, plus 1 additional post on a positive nonpandemic topic to promote engagement. Posts in the 3 groups had the same messages but differed by links to information from government agencies, near-peer parents, or news media in the post. Mothers reported on social distancing behavior and COVID-19 vaccine intentions for self and daughter, theoretic mediators, and covariates in baseline and 3-, 6-, and 9-week postrandomization assessments. Views, reactions, and comments related to each post were counted to measure engagement with the messages.

**Results:**

Nearly all mothers (n=298, 98.3%) remained in the Facebook private groups throughout the 9-week trial period, and follow-up rates were high (n=276, 91.1%, completed the 3-week posttest; n=273, 90.1%, completed the 6-week posttest; n=275, 90.8%, completed the 9-week posttest; and n=244, 80.5%, completed all assessments). In intent-to-treat analyses, social distancing behavior by mothers (b=–0.10, 95% CI –0.12 to –0.08, *P*<.001) and daughters (b=–0.10, 95% CI –0.18 to –0.03, *P*<.001) decreased over time but vaccine intentions increased (mothers: b=0.34, 95% CI 0.19-0.49, *P*<.001; daughters: b=0.17, 95% CI 0.04-0.29, *P*=.01). Decrease in social distancing by daughters was greater in the near-peer source group (b=–0.04, 95% CI –0.07 to 0.00, *P*=.03) and lesser in the government agency group (b=0.05, 95% CI 0.02-0.09, *P*=.003). The higher perceived credibility of the assigned information source increased social distancing (mothers: b=0.29, 95% CI 0.09-0.49, *P*<.01; daughters: b=0.31, 95% CI 0.11-0.51, *P*<.01) and vaccine intentions (mothers: b=4.18, 95% CI 1.83-6.53, *P*<.001; daughters: b=3.36, 95% CI 1.67-5.04, *P*<.001). Mothers’ intentions to vaccinate self may have increased when they considered the near-peer source to be not credible (b=–0.50, 95% CI –0.99 to –0.01, *P*=.05).

**Conclusions:**

Decreasing case counts, relaxation of government restrictions, and vaccine distribution during the study may explain the decreased social distancing and increased vaccine intentions. When promoting COVID-19 prevention, campaign planners may be more effective when selecting information sources that audiences consider credible, as no source was more credible in general.

**Trial Registration:**

ClinicalTrials.gov NCT02835807; https://clinicaltrials.gov/ct2/show/NCT02835807

## Introduction

### Background

To control the COVID-19 pandemic, the Centers for Disease Control and Prevention (CDC) has advised Americans to practice nonpharmaceutical interventions (NPIs; eg, social distancing, masking, and reduced group participation) and federal and state governments have mounted an unprecedented biomedical endeavor to develop and distribute vaccines [1-3]. NPIs are feasible, and social distancing and mask wearing reduce SARS-CoV-2 transmission [4-9]. Attention to prevention measures remains necessary because use of NPIs has declined and governments have relaxed restrictions [10-12]; even though vaccines are not universally accepted [13,14], individuals need to be revaccinated [15,16]; and groups that do not support vaccination are undermining confidence in COVID-19 vaccines [17,18].

In this study conducted from January to March 2021, we tested the impact of an intervention comprising social media posts promoting COVID-19 NPIs and vaccine intentions and compared 3 different types of information sources highlighted in the posts. In January 2021, COVID-19 case rates were high (7-day moving average=165,974 cases on January 25) [19] and NPIs were strongly recommended or mandated [20,21]. However, cases had declined substantially by March 2021 (7-day moving average=59,986 cases on March 26) [19] and some states were relaxing NPI advice and restrictions [20-22]. Two vaccines had been approved by January 2021 and a third in March 2021. Mass vaccination began during the intervention [22], but most states were still restricting vaccination to middle-age and older adults, with only 32% of American adults having received at least 1 dose at the end of March 2021 [23].

### Role of Social Media in the COVID-19 Pandemic

Social media has played a large role in disseminating pandemic information, but it has also been used to spread misinformation [3,24], such as lack of severity of COVID-19, false virus transmission methods, ineffective prevention and diagnostic methods, unproven/pseudoscience treatments, risks from testing and face masks, and other conspiracy theories [25-28]. Misinformation has also spread about the COVID-19 vaccine, such as claims that vaccine safety was compromised by the rush to market, that the low risk from COVID-19 and effective prevention and treatment make vaccines less necessary, and that variation in the amount and length of effectiveness indicates vaccines are not useful [13,17]. Lower uptake of vaccines in general and lower COVID-19 vaccine intentions have been related to misinformation, unwarranted safety concerns, and conspiracies on social media, as has the practice of NPIs [29,30]. Thus, efforts are needed to promote COVID-19 prevention measures and correct misinformation on social media through fact checking and corrections, counternarratives, peer correction, coherence/credibility appeals, and digital and media literacy [31-38].

### Impact of Sources for COVID-19 Information

The Extended Parallel Process Model (EPPM) of risk communication [39], an extension of protection motivation theory (PMT) [40,41], has explained mitigation behaviors in past pandemics, uptake of other vaccines [42-44], and COVID-19 pandemic responses [45]. It holds that the credibility of information sources influences the effectiveness of health messages [46]. High-credibility sources make it difficult for campaign audiences to derogate sources in order to decrease fear from risk information about COVID-19. In this way, messages from high-credibility sources motivate individuals to take actions that reduce risk with NPIs and vaccines.

We experimentally varied 3 types of sources, popular for information about the pandemic [47-49], that can vary in credibility (eg, trustworthiness and accuracy) in the social media posts on COVID-19: government agency, near-peer parents, and news media. Government health authorities are trusted sources of COVID-19 information for many (but not all) people [50,51], with nongovernmental content and unverifiable sources seen as less trustworthy, especially when posted on social media platforms [52,53]. A cross-sectional study of COVID-19 information sources found that attention to government sources is linked to greater COVID-19 knowledge [50]. Content shared on social media from (perceived) knowledgeable peers can have credibility and impact through identification processes based on similarity [54-57]. Peers (eg, friends, family, and work colleagues) have also been an often-used source of information about COVID-19, although they are not always as trusted as government and news media sources [48,51]. Consumers evaluate the credibility of both the source and message content of news media [58]. One study found that exposure to news media reduces conspiracy theories and misinformation beliefs regarding COVID-19 [59], but another reported that COVID-19 knowledge is lower among individuals who have greater trust in these sources [50]. The availability of a variety of information sources can elevate risk perceptions and fear; create information overload, anxiety, stress, and other negative psychological states; and possibly cause people to avoid information [45,47,48,60].

### Hypothesis and Research Questions

This study was conducted with mothers of daughters aged 14-17 years who had participated in a previous trial on adolescent health. Mothers are an important audience for a COVID-19 prevention campaign because (1) mothers are often a primary decision maker for health and vaccination in families [61-63] and (2) parents use social media to track public health issues, share information, and seek advice [64]. The study tested the following primary hypothesis (H):

- H1: Mothers will report increased COVID-19 social distancing behaviors and vaccine intentions over the intervention period from baseline across 3 follow-up measures.

Posts also addressed theoretic antecedents of prevention behaviors prominent in the EPPM and social cognitive theory (SCT) [65]. In addition, whether mothers communicated with daughters about COVID-19 NPIs and vaccines was assessed because mother–daughter communication has influenced health behaviors of adolescent and young adult daughters in past research [66-68].

- H2: Mothers will report improved theoretic antecedents (perceived risk, self-efficacy, and response efficacy and cost) and mother–daughter communication about COVID-19 over the course of the intervention from baseline across 3 follow-up measures.

Analyses explored research questions asking whether the rate of change in social distancing, vaccine intentions, theoretic antecedents, and mother–daughter communication differed among the 3 types of information sources or by engagement with the social media messages.

## Methods

### Sample

Mothers were recruited to the study from a sample who had previously participated in a trial evaluating a social media campaign to prevent teen daughters from indoor tanning. In the original trial, mothers were recruited using community-based strategies (eg, schools, community events) and from the Qualtrics survey panel and met the following inclusion criteria: (1) having a daughter aged 14-17 years, (2) living in 1 of 34 states without a complete ban on indoor tanning (IT) by minors, (3) reading English, (4) having a Facebook account and logging in at least once per week, and (5) willing to “friend” the project’s community manager to join a private Facebook group. A detailed description of trial procedures has been published elsewhere [69,70]. In January 2021, 830 mothers were recontacted by email, invited to enroll in the current study that was described as a private group related to how mothers and daughters cope with the COVID-19 pandemic. Daughters were not enrolled in this study.

### Experimental Design

Mothers were enrolled in a randomized pretest–posttest single-factor-design study with 4 assessments. After completing the baseline survey, mothers were randomly assigned to 1 of 3 experimental conditions that varied in the type of sources in the posts (government health agencies, near-peer parents, or news media) using a routine in Qualtrics survey software. Mothers “friended” the project community manager and were added to a Facebook private group for their assigned condition. As all mothers received experimental social media messages, they were blind to experimental manipulation of the information source. Study staff, other than the community manager and project manager, were blinded, too. The private groups prevented contamination between treatment groups while delivering the social media messages and made it possible to record engagement. Randomization controlled for background secular exposure to information in social media and other sources about COVID-19. Mothers received a series of Facebook posts for 9 weeks starting after randomization from January 25 to March 26, 2021. Each post contained text with a link to related information from 1 of the 3 types of sources. Mothers stayed in the groups for 9 weeks, completing online posttests at 3, 6, and 9 weeks postrandomization. After the intervention, 30 (9.9%) of 303 mothers were randomly selected to participate in focus groups, where they were asked what they liked most and least about the Facebook group and what they learned. A priori statistical power calculations via a Monte Carlo study in Mplus and with the *powerlmm* package [71] in R software (R Foundation for Statistical Computing) indicated that an initial sample size of 300 mothers (100 per condition) would have 0.90 power to detect a moderate-size rate of increase in vaccine intention (Cohen d=0.50). Retention was achieved by alerting mothers to upcoming posttests and compensating mothers for assessments (US $20 for baseline, US $10 for each posttest). Mothers also received 1 raffle entry for every survey completed in drawings for 20 US $100 gift cards after the final posttest.

### Ethical Considerations

Mothers provided informed consent online before completing the baseline survey. The study procedures were approved by the Western Institutional Review Board (1-872442-1).

### Intervention

The intervention contained 45 Facebook posts related to COVID-19 (5, 11.1%, per week) designed by the research team based on the EPPM [39] and SCT [65]. Posts addressed 4 topics: the 2 primary outcomes (NPIs and COVID-19 vaccination), digital and media literacy, and mother–daughter communication. These topics were rotated across weekdays by week to ensure that all topics had the same likelihood of being viewed. Posts on digital and media literacy were included to combat misinformation related to NPIs and vaccines by addressing source credibility, fact checking, lateral reading, sharing of posts with family/friends, social media algorithms, rebutting of misinformation, and deep fake videos [72-74]. Posts encouraged mothers to talk with teen daughters about the pandemic and promote prevention behavior [66-68] and sought to improve this communication by teaching skills, such as active listening, self-disclosure, empathy, and conflict management. Across these topics, posts addressed theoretic antecedents, including risk from COVID-19 (ie, severity and susceptibility), self-efficacy and response efficacy of NPIs and vaccination, descriptive norms for NPIs and vaccination, behavioral capability (knowledge of risks of COVID-19 and skills to practice NPIs), and observational learning (stories about dangers of COVID-19 and skills related to NPIs, vaccination, and family communication). To increase mothers’ engagement, posts encouraged mothers to react to (eg, like) and comment on posts, for example, by asking a question to solicit the mothers’ own experience and opinions on a topic. Additionally, 12 posts provided study information or were aimed at engaging mothers with holiday plans, favorite books, family traditions, and recipes.

Each experimental post contained the same content in all 3 groups. The experimental manipulation of information sources was accomplished by linking each message in the posts to additional online content (eg, articles, blog posts, infographics, or videos) from either a government agency (eg, the CDC or the World Health Organization [WHO]), a near-peer parent, or news media. For the near-peer parent group, information was sourced primarily from Twitter, Instagram, Facebook, TikTok, and parenting blog posts or magazines. Near-peer parents were predominantly women. The term “near-peer” was used to reflect that these sources were similar to the participants, being obviously parents (although a few were female journalists, college professors, or nurses), and were selected to be close to the age of the sample (range 28-64 years, mean 42.7, SD 6.7). However, these sources were unlikely to be known personally by participants, as might be a “peer.” For news media, content was sourced from 22 media organizations that focused on delivering news to the general public or a target public. Since individuals can differ in the credibility they assign to various news media, we selected content from news media that ranged from moderately conservative (eg, Fox News and the *New York Post*) to middle-of-the-road (eg, USA Today and *Newsweek*) to moderately liberal (eg, *Washington Post* and ABC), as ranked by All Sides Media Bias [77]. The research team confirmed that all links and content from information sources were accurate. Some of the content from the source was embedded in the experimental post (eg, infographic or screenshot), but a link was always provided to the information source.

Posts were developed by the investigators using an agile development process to reflect the rapidly changing pandemic information environment and ensure content was timely and relevant. Mothers (n=30, 9.9%) participated in virtual focus groups before and during the intervention to review and provide feedback on sample posts. Initially, 2 weeks of posts were prepared, after which new posts were developed weekly. All posts were reviewed by 4 of the investigators (authors DB, BW, WGW, SP), the project manager, and the community manager for readability, theoretical principles, accuracy, and information source prior to posting.

Posts were scheduled by the community manager. They appeared at 10:00 a.m. on Monday, Wednesday, and Friday and 7:00 p.m. on Tuesday and Thursday (1 post per day). Posting times were based on analytics from our prior study regarding the most popular times to view posts [69]. The initial post welcomed participants to the group, invited them to join in discussing the posts, and asked them to be respectful of other group members during discussions and to maintain the privacy of other participants when they communicated about content in the posts with family and friends outside the group. Posts on the 4 topics (NPIs, vaccination, digital and media literacy, and mother–daughter communication) appeared each week (1 post on each of the 3 topics and 2 posts on 1 topic in a week; topics with 2 posts were rotated across the weeks). On Wednesdays, an additional engagement post was published (n=12) to balance the seriousness of the pandemic topics and help keep mothers engaged. The community manager followed a protocol to monitor mothers’ reactions and comments to each post and respond to any uncertainty or misinformation or requests for additional information from mothers. Responses had a respectful, empathy-driven, reflective-listening approach toward the mothers [76] that acknowledged the mothers’ comments, advised them to follow local and national COVID-19 guidelines, and included links to government agencies, professional groups (eg, the American Diabetes Association), and news media.

### Measures

All measures were self-reported by mothers and collected using Qualtrics survey software (see Multimedia Appendix 1).

#### Primary Outcomes

The primary outcomes, assessed at pretest and all posttests, were social distancing behaviors by self and daughters (self: *α*=.76 [baseline], .76 [week 3], .79 [week 6], .76 [week 9]; daughters: *α*=.76 [baseline], .72 [week 3], .78 [week 6], .78 [week 9]) [45,77,78] and mothers’ intentions to vaccinate self and daughters for COVID-19 [79]. The vaccine intention questions were modified to use a 0-100 scale (0=definitely would not get the vaccine to 50=unsure whether I would get the vaccine to 100=definitely would get the vaccine) to maximize heterogeneity in responses and avoid forcing participants to choose among a finite set of categories. The intention scores were bimodal, so we divided responses into 5 categories based on the raw data plots: 1=0-20, 2=21-40, 3=41-60, 4=61-80, and 5=81-100. In the 9-week posttest, mothers were also asked whether they had received a COVID-19 vaccination; if vaccinated, mothers’ vaccine intention was coded as 100.

#### Theoretic Antecedents

Theoretic antecedents from the EPPM and SCT were assessed, including perceived risk of COVID-19 (severity *α*=.86, susceptibility *α*=.72), self-efficacy for NPIs [45,80] and COVID-19 vaccination (*α*=.72-.73 [baseline], .59-.67 [week 3], .69-.67 [week 6], .58-.62 [week 9]) [81], and response efficacy (*α*=.91 [baseline], .92 [week 3], .80 [week 6], .89 [week 9]) response cost (*α*=.71 [baseline], .74 [week 3], .70 [week 6], .70 [week 9]) for COVID-19 NPIs [45].

#### Mother–Daughter Communication

Mother–daughter communication about COVID-19 was measured using a scale modified from the original trial [69,70], which asked whether they had discussed the 7 topics about COVID-19 with their daughters (*α*=.70 [baseline], .75 [week 3], .83 [week 6], .80 [week 9]).

#### Source Credibility

The credibility of the government agency, near-peer parents, and news media for COVID-19 information was assessed in 2 ways. At baseline, mothers rated the credibility of these 3 information sources on trustworthy, accurate, and bias (*α*=.79 [government], .76 [near-peer parent], .55 [news media]) [82]. In each posttest, mothers used these same items to rate 1-2 posts from their assigned group in the preceding 3 weeks (*α*=.60 [week 3], .64 [week 6], .63 [week 9, media literacy], .77 [week 9, mother–daughter communication]). Posts on social distancing (week 3), vaccination (week 6), media literacy (week 9), and mother–daughter communication (week 9) were presented at random.

#### Media Use

Mothers’ media use was assessed at baseline. Mothers were asked about exposure to COVID-19 messages in the media (*α*=.91) [83]. They also reported the number of hours in a typical day they used any media to obtain news and information and used any media to inform themselves about COVID-19 [84]. Mothers completed measures on COVID-19 information overload (*α*=.76) and excessiveness (*α*=.60).

#### Mothers’ Characteristics

Finally, individual differences among mothers on political leaning (conservative, middle-of-the-road, or liberal), history of COVID-19 infection (Do you believe you had COVID-19, and have you ever received a test to check for COVID-19 infection?) [78], vaccination antecedents (*α*=.82) [85], demographics (ie, race, Hispanic ethnicity, age, and education), urbanization of home county (from US Census), and health insurance status of self and daughter [86] were obtained from the original trial or the baseline survey.

#### Engagement With Social Media Messages

Engagement with the Facebook posts was recorded in 3 ways. Mothers’ reactions (eg, like, love, wow, angry, and sad) and comments on all posts were extracted in the identified format using a customized program and counted. The number of views per post was recorded. Mothers reported whether they read posts on COVID-19, whether they felt connected to the group, and whether they shared/communicated about the posts on COVID-19 in the final posttest.

#### Acceptability of the Facebook Group

Finally, acceptability of the social media messages in the Facebook private group was evaluated in postintervention focus groups via 3 questions:

- What did you like most about the Facebook group?
- What did you like least about the Facebook group?
- What did you learn from the Facebook group?

Recordings of focus group discussions were reviewed and coded using a conventional content analysis protocol [87]. Two trained coders independently classified responses, and discussion was used to achieve consensus on disagreements. Interrater reliability was adequate (*κ*=0.78-0.87) [88]. We summarized the frequency of themes.

### Statistical Analysis

Two sets of analyses were conducted to test the prespecified hypotheses and research questions. In the first set, a series of mixed effects growth models were used to model change in each of 4 primary outcomes (mothers’ reports of social distancing behavior and vaccine intentions by self and daughters), 8 theoretic antecedent outcomes (perceived risk [severity and susceptibility], response efficacy and cost of NPIs, self-efficacy for NPIs and vaccination [self and daughters]), and mother–daughter communication in the hypotheses. Each outcome (measured over 4 occasions) was regressed on time (centered at 9 weeks), effect codes for treatment, and time-by-effect-code interactions. Random effects for the intercept and slope were included and specified to correlate. With the effect codes, estimates for the intercept (centered at week 9) and slope for time (rate of change in the outcome over time) represented the average of these estimates for the 3 conditions, rather than 1 single reference group as with dummy codes. An ordinal mixed effects model was fit for intentions to vaccinate, and a linear mixed effects model was fit for the other 13 outcomes. In the second set of analyses, 4 mixed effects models for social distancing behavior and vaccine intentions were examined to test the moderating effect of engagement with the social media feed to test the second research question. All models included all possible interactions between time, condition, and the moderator, with the treatment condition represented by effect codes. Therefore, simple effects for time and the moderators represented the average effect across the 3 conditions.

Next, a set of exploratory analyses were performed. Analyses fit mixed effects models to explore 4 additional possible moderators: baseline source credibility, COVID-19 media consumption, political leaning on social distancing behavior and vaccine intentions, and baseline vaccine intentions on follow-up vaccine intentions. Mothers’ averaged interim credibility ratings of 4 posts from the Facebook private groups were examined as a moderator of treatment effects on social distancing behavior and vaccine intentions measured at week 9. A linear model was fit for social distancing behaviors and an ordinal regression model for intentions, regressing them on treatment (represented as 2 effect codes), post credibility, interaction of treatment and post credibility, baseline rating of credibility of the assigned treatment condition, and baseline rating of the outcome.

## Results

### Profile of the Sample

Overall, 303 mothers were enrolled (n=100, 33.0%, in the government agency group; n=99, 32.7%, in the near-peer parent group; n=104, 34.3%, in the news media group). Mothers were middle aged (range 28-64 years); well educated, with 160 (55.7%) completing college; and moderately affluent, with 150 (56.4%) having incomes over US $80,000 (see Tables 1-3). Nearly all were non-Hispanic White, because the original trial aimed at preventing indoor tanning. Mothers had diverse political leaning, and the majority lived in states with Republican governors. About 1 (22%) in 5 mothers believed that they had COVID-19 in the past, and nearly half (n=155, 51.3%) had been tested (n=30, 9.9%, had tested positive). At baseline, 199 (65.7%) of the participants lived in states with a mask mandate, and most states were limiting vaccination to older individuals (aged 46.1 years on average). There were no statistically significant differences between the participants’ characteristics by treatment group at baseline.

**Figure 1:**
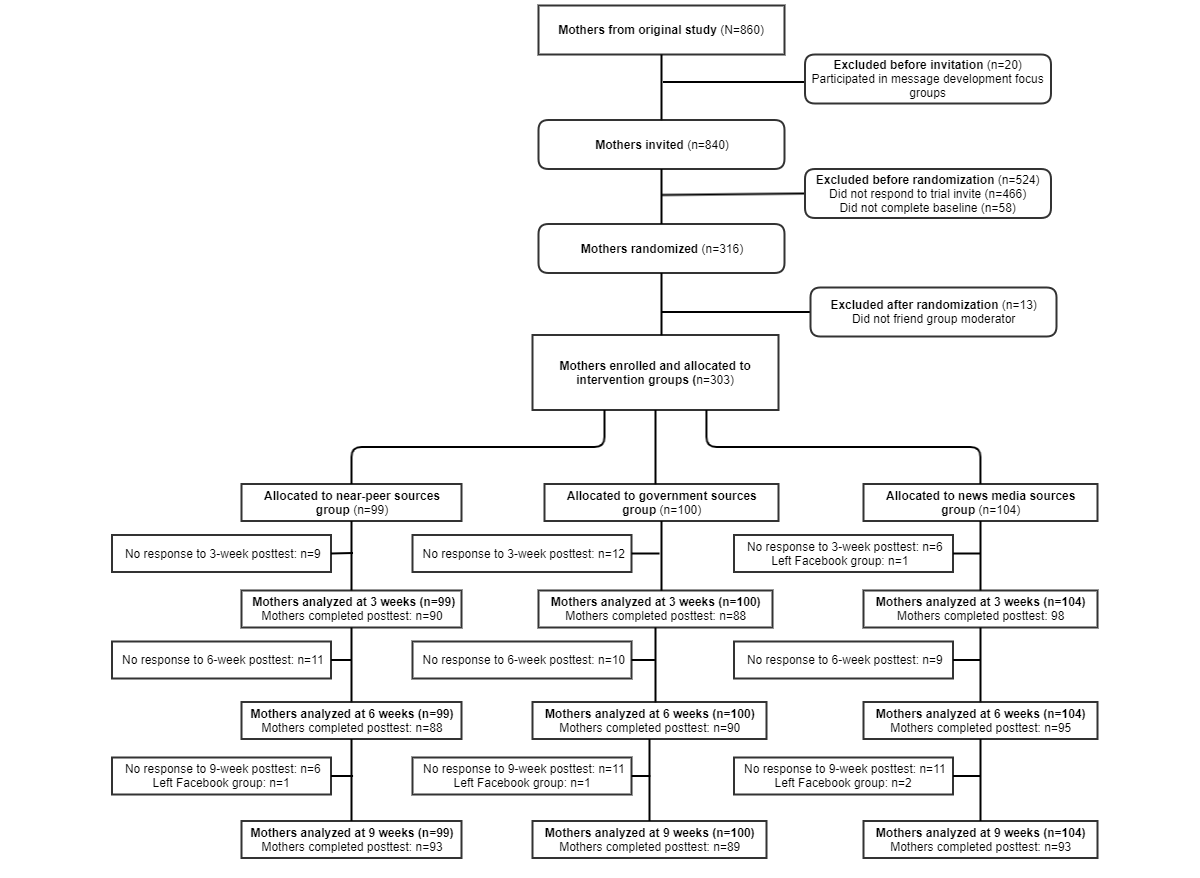
CONSORT diagram for trial. CONSORT: Consolidated Standards of Reporting Trials.

The retention of mothers was high. Nearly all mothers (n=298, 98.3%) remained in the Facebook private groups throughout the 9-week period (ie, did not actively “unfriend” themselves from the private group). Similarly, 276 (91.1%) completed the 3-week posttest, 273 (90.1%) completed the 6-week posttest, and 275 (90.8%) completed the 9-week posttest, while 244 (80.5%) completed all assessments; see the Consolidated Standards of Reporting Trials (CONSORT) diagram in Figure 1.

Mothers appeared to engage with the 57 messages posted to each Facebook private group. On average, mothers viewed over 35 posts (government mean 36.79 [SD 20.45], near-peer parents mean 37.30 [SD 8.99], news media mean 40.38 [SD 24.20]) and posted reactions or comments on over 10 of the posts (government mean 11.46 [SD 18.57], near-peer parents mean 10.23 [SD 16.51], news media mean 11.41 [SD 17.37]).

**Table 1:** Demographic characteristics of participants by treatment group.

Characteristics		Overall (N=303)	Treatment group		
			Government agency (n=100)	Near-peer parents (n=99)	News media (n=104)
Age (years), mean (SD)		42.8 (6.7)	42.7 (6.6)	42.8 (6.8)	42.8 (6.8)
**Ethnicity, n (%)**					
	Hispanic	19 (6.3)	10 (10.0)	4 (4.0)	5 (4.8)
	Non-Hispanic	284 (93.7)	90 (90.0)	95 (96.0)	99 (95.2)
**Race, n (%)**					
	American Indian/Alaska Native	3 (1)	1(0.3)	1 (0.3)	1 (0.3)
	Asian	4 (1.3)	0 (0)	4 (4.0)	0 (0)
	Black/African American	23 (7.6)	7 (7)	8 (8.1)	8 (7.7)
	White	264 (87.1)	90 (90)	83 (83.8)	91 (87.5)
	Other	5 (1.7)	1 (1.0)	1 (1.0)	3 (2.9)
	More than 1 race	4 (1.3)	2 (2.0)	1 (1.0)	1 (1.0)
**Education, n (%)**					
	High school or less	22 (7.7)	6 (6.2)	5 (5.3)	11 (11.3)
	Some education beyond high school	105 (36.6)	35 (36.5)	39 (41.5)	31 (32.0)
	4-year college graduate	81 (28.2)	26 (27.1)	26 (27.7)	29 (29.9)
	Postgraduate education	79 (27.5)	29 (30.2)	24 (25.5)	26 (26.8 )
**Total annual household income (US $), n (%)**					
	20,000 or less	13 (4.9)	2 (2.3)	6 (7.0)	5 (5.4)
	20,001-40,000	32 (12.0)	12 (13.6)	7 (8.1)	13 (14.1)
	40,001-60,000	38 (14.3)	9 (10.2)	15 (17.4)	14 (15.2)
	60,001-80,000	33 (12.4)	16 (18.2)	7 (8.1)	10 (10.9)
	80,001-100,000	49 (18.4)	19 (21.6)	14 (16.3)	16 (17.4)
	More than 100,000	101 (38.0)	30 (34.1)	37 (43.0)	34 (37.0)

**Table 2:** COVID-19 prevention and history characteristics of participants by treatment group.

Characteristics		Overall (N=303)	Treatment group		
			Government agency (n=100)	Near-peer parents (n=99)	News media (n=104)
**Statewide mask mandate in state of residence, n (%)**					
	Yes	199 (65.7)	71 (71.0)	57 (57.6)	71 (68.3)
	No	104 (34.3)	29 (29.0)	42 (42.4)	33 (31.7)
Age eligibility for COVID-19 vaccine (years), mean (SD)		46.1 (17.7)	46.1 (17.7)	47.7 (17.2)	43.9 (18.2)
**Have you ever received a test to check for COVID-19 infection?, n (%)**					
	Yes, tested positive	30 (9.9)	12 (12.0)	10 (10.2)	8 (7.7)
	Yes, tested negative	123 (40.7)	46 (46.0)	40 (40.8)	37 (35.6)
	Yes, still waiting for test results	2 (0.7)	1 (1.0)	0 (0.0)	1 (1.0)
	No	147 (48.7)	41 (41.0)	48 (49.0)	58 (55.8)
**Do you believe that you have had COVID-19?, n (%)**					
	Yes	67 (22.2)	25 (25.0)	22 (22.4)	20 (19.2)
	No	197 (65.2)	63 (63.0)	63 (64.3)	71 (68.3)
	I don’t know	38 (12.6)	12 (12.0)	13 (13.3)	13 (12.5)

**Table 3:** Political ideology characteristics of participants by treatment group.

Characteristics			Overall (N=303)	Treatment group				
				Government agency (n=100), n (%)		Near-peer parents (n=99), n (%)		News media (n=104), n (%)
**Political leaning**								
	Conservative	72 (24.4)		25 (25.2)	25 (25.8)		22 (22.2)	
	Middle-of-the-road	148 (50.2)		54 (54.6)	48 (49.5)		46 (46.5)	
	Liberal	75 (25.4)		20 (20.2)	24 (24.7)		31 (31.3)	
**Political affiliation of governor of state of residence**								
	Democratic	115 (38.0)		44 (44.0)	31 (31.3)		40 (38.5)	
	Republican	188 (62.0)		56 (56.0)	68 (68.7)		64 (61.5)	

### Hypothesis 1 Test: Change in Social Distancing and Vaccine Intentions

At baseline, most mothers reported that they and their daughters were engaging in a moderate to high levels of social distancing (Table 4). Mothers’ reports of social distancing by both themselves and daughters decreased over time when examining all 3 posttests relative to baseline (Table 5), disconfirming H1.

About half of the mothers had high vaccine intentions for themselves and their daughters, but up to one-quarter expressed low vaccine intentions (Table 4). Vaccine intentions for self and daughters increased over time (Table 5), supporting H1. However, vaccine intentions were bimodally distributed, with large groups of mothers consistently indicating low (<20.00 likelihood) and high (80.00 likelihood) intentions across all 4 time points. Thus, baseline vaccine intentions were split into 3 groups (low<20.00, moderate=20.00-79.00, and high80.00 likelihood) and tested as a moderator of change in the 5-level vaccine intention measure in the 3 posttests. There was a statistically significant improvement in vaccine intentions for self (b=0.76, 95% CI 0.31-1.21, *P*<.01) and daughters (b=0.48, 95% CI 0.06-0.89, *P*=.02) over time among mothers with moderate intentions at baseline. Likewise, there was a statistically significant increase in vaccine intention for self (b=9.21, 95% CI 6.60-11.82, *P*<.001) and daughters (b=5.51, 95% CI 3.78-7.23, *P*<.001) by the 9-week posttest among mothers with high baseline intentions. Mothers with low baseline vaccine intentions reported lower vaccine intention for self (b=–5.99, 95% CI –8.03 to –3.95, *P*<.001) and daughters (b=–4.83, 95% CI –6.69 to –2.97, *P*<.01) in the 9-week posttest.

**Table 4:** Percentage of mothers (N=303) reporting social distancing and vaccine intentions for themselves and daughters at baseline.

Ratings		Themselves, n (%)	Daughters, n (%)
**Social distancing**			
	Low (rating=1.00-2.33)	12 (4.0)	8 (2.6)
	Moderate (rating=2.34-3.66)	104 (34.3)	117 (38.7)
	High (rating=2.67-5.00)	187 (61.7)	178 (58.7)
**Intention to vaccinate**			
	Low (likelihood=0-20)	73 (24.5)	67 (22.6)
	Moderate (likelihood=21-80)	73 (24.5)	94 (31.6)
	High (likelihood=81-100)	152 (51.0)	136 (45.8)

**Table 5:** Results of regression analyses of a change in primary outcomes and theoretic mediators over time from baseline across 3-, 6-, and 9-week posttests.

		b	95% CI	*P* value
**Social distancing**				
	Mother	–0.10	–0.12 to –0.08	<.001
	Daughter	–0.10	–0.12 to –0.03	<.001
**Intent to vaccinate**				
	Mother	0.34	0.19-0.49	<.001
	Daughter	0.17	0.04-0.29	.01
Self-efficacy for NPIs^a^		0	–0.03 to 0.03	.96
**Self-efficacy for vaccination**				
	Mother	0.08	0.05-0.12	<.001
	Daughter	0.05	0.01-0.08	<.01
Response efficacy for NPIs		0.01	–0.02 to 0.03	.59
Response cost for NPIs		–0.03	–0.05 to 0.00	.02
**Perceived risk**				
	Severity	0.04	0.01-0.07	.01
	Susceptibility	–0.03	–0.06 to 0.00	.04
Mother–daughter communication		–0.02	–0.06 to 0.01	.16

^a^NPI: nonpharmaceutical intervention.

### Hypothesis 2 Test: Change in Theoretic Antecedents and Mother–Daughter Communication

Several theoretic antecedents improved over time (Table 4), largely supporting H2. Specifically, self-efficacy for vaccination of self and daughters increased, and response costs for NPIs decreased. There was also some evidence that perceived risk increased over time, particularly with the severity of COVID-19 increasing over time; however, perceived susceptibility declined over time. By contrast, self-efficacy and response efficacy for NPIs did not change, nor did mother–daughter communication (Table 5), contrary to the hypothesis.

### Differences Among Information Sources

#### Effect of Treatment Group

Only 1 outcome was moderated by the experimental manipulation of information sources. The decline in social distancing by daughters over time was greater when mothers were in the near-peer parents group (b=–0.04, 95% CI –0.07 to 0.00, *P*=.03) and lesser when mothers were in the government agency group (b=0.05, 95% CI 0.02-0.09, *P*=.003); see Table 6. Interactions between treatment group and time were not statistically significant for social distancing by mothers (near-peer parents: b=–0.01, 95% CI –0.03 to 0.02, *P*=.66; government agency: b=0.01, 95% CI –0.02 to 0.04, *P*=.51) and mother–daughter communication (near-peer parents: b=–0.03, 95% CI –0.08 to 0.02, *P*=.22; government agency: b=0.02, 95% CI –0.03 to 0.06, *P*=.51); see Table 7.

The information source moderated the improvement in mothers’ own vaccine intentions in the analysis treating baseline vaccine intentions as a moderator. The increase in mothers’ vaccine intentions among those who had high intentions at baseline was attenuated in the government agency source condition, both for change across all 3 posttests (b=–1.47, 95% CI –2.74 to –0.20, *P*=.02) and at the 9-week posttest (b=–3.17, 95% CI –5.91 to –0.43, *P*=.02).

**Table 6:** Means (SD) of social distancing behavior and vaccine intention measures by treatment condition and time of assessment.

Outcome and source			Baseline		3-week posttest		6-week posttest		9-week posttest
**Mothers’ social distancing**									
	Government agency	3.90 (0.77)		3.80 (0.80)		3.72 (0.83)		3.62 (0.79)	
	Near-peer parents	3.87 (0.76)		3.74 (0.84)		3.67 (0.89)		3.56 (0.86)	
	News media	3.97 (0.68)		3.84 (0.76)		3.75 (0.83)		3.65 (0.86)	
**Daughters’ social distancing**									
	Government agency	3.77 (0.70)		3.79 (0.74)		3.68 (0.75)		3.66 (0.75)	
	Near-peer parents	3.86 (0.72)		3.74 (0.71)		3.58 (0.83)		3.46 (0.87)	
	News media	3.98 (0.72)		3.82 (0.76)		3.68 (0.84)		3.64 (0.89)	
**Vaccine intentions for self**									
	Government agency	3.46 (1.78)		3.38 (1.80)		3.53 (1.75)		3.69 (1.68)	
	Near-peer parents	3.70 (1.64)		3.63 (1.71)		3.82 (1.64)		3.86 (1.65)	
	News media	3.70 (1.65)		3.66 (1.75)		3.76 (1.74)		3.80 (1.72)	
**Vaccine intentions for daughters**									
	Government agency	3.49 (1.71)		3.52 (1.72)		3.56 (1.66)		3.71 (1.61)	
	Near-peer parents	3.60 (1.59)		3.50 (1.69)		3.60 (1.63)		3.77 (1.62)	
	News media	3.66 (1.64)		3.66 (1.65)		3.75 (1.66)		3.74 (1.60)	

**Table 7:** Means (SD) of secondary outcome measures by treatment condition and time of assessment.

Outcome and source		Baseline		3-week posttest		6-week posttest		9-week posttest
**Perceived risk: severity**								
	Government agency	4.34 (0.85)	4.42 (0.80)		4.42 (0.88)		4.52 (0.72)	
	Near-peer parents	4.33 (0.89)	4.28 (0.99)		4.34 (0.79)		4.46 (0.80)	
	News media	4.36 (0.70)	4.49 (0.74)		4.45 (0.78)		4.44 (0.80)	
**Perceived risk: susceptibility**								
	Government agency	3.56 (0.86)	3.46 (0.99)		3.44 (0.90)		3.54 (0.96)	
	Near-peer parents	3.49 (0.96)	3.37 (0.98)		3.43 (0.81)		3.40 (0.92)	
	News media	3.50 (0.76)	3.56 (0.77)		3.42 (0.81)		3.28 (0.89)	
**Response efficacy of NPIs^a^**								
	Government agency	4.48 (0.66)	4.56 (0.68)		4.42 (0.63)		4.57 (0.62)	
	Near-peer parents	4.51 (0.76)	4.56 (0.74)		4.41 (0.76)		4.53 (0.70)	
	News media	4.55 (0.71)	4.54 (0.53)		4.68 (0.50)		4.54 (0.66)	
**Response cost for NPIs**								
	Government agency	4.43 (0.65)	4.45 (0.68)		4.40 (0.72)		4.39 (0.63)	
	Near-peer parents	4.49 (0.69)	4.45 (0.73)		4.41 (0.72)		4.42 (0.80)	
	News media	4.38 (0.78)	4.37 (0.80)		4.40 (0.78)		4.26 (0.90)	
**Self-efficacy for NPIs**								
	Government agency	4.35 (0.67)	4.40 (0.64)		4.34 (0.73)		4.35 (0.69)	
	Near-peer parents	4.28 (0.79)	4.22 (0.80)		4.27 (0.80)		4.22 (0.79)	
	News media	4.19 (0.84)	4.28 (0.80)		4.30 (0.80)		4.27 (0.82)	
**Self-efficacy for vaccinating mothers**								
	Government agency	3.88 (1.01)	3.95 (1.01)		4.03 (1.10)		4.19 (0.97)	
	Near-peer parents	4.15 (0.86)	4.07 (0.94)		4.19 (0.90)		4.32 (0.79)	
	News media	3.89 (1.10)	4.00 (1.03)		4.16 (1.03)		4.15 (0.97)	
**Self-efficacy for vaccinating daughters**								
	Government agency	3.83 (0.98)	3.80 (0.99)		3.98 (1.10)		4.06 (1.01)	
	Near-peer parents	4.02 (0.89)	3.95 (1.03)		4.06 (0.91)		4.05 (0.90)	
	News media	3.85 (1.04)	3.93 (1.04)		3.99 (1.04)		3.95 (1.03)	
**Mother–daughter communication about COVID-19**								
	Government agency	3.50 (0.86)	3.28 (0.98)		3.39 (1.08)		3.43 (1.06)	
	Near-peer parents	3.65 (0.82)	3.42 (0.97)		3.51 (1.06)		3.44 (1.08)	
	News media	3.62 (0.85)	3.45 (0.98)		3.50 (1.09)		3.57 (0.96)	

^a^NPI: nonpharmaceutical intervention.

#### Moderation by Perceived Credibility of the Assigned Information Source

Approximately one-third of the mothers considered the assigned information source to be credible in general at baseline (government agency: n=100, 33.0%; near-peer parents: n=99, 32.7%; news media: n=104, 34.3%). Perceived credibility was associated with an increase in social distancing and vaccine intentions over time. Mothers who rated the assigned information source as credible reported greater social distancing for self (b=0.29, 95% CI 0.09-0.49, *P*<.01) and daughters (b=0.31, 95% CI 0.11-0.51, *P*<.01) and higher vaccine intentions for self (b=4.18, 95% CI 1.83-6.53, *P*<.001) and daughters (b=3.36, 95% CI 1.67-5.04, *P*<.001) at the 9-week posttest. However, these improvements in social distancing and vaccine intentions associated with source credibility were attenuated substantially in the near-peer parents condition (credibility × condition: social distancing, self: b=–0.41, 95% CI –0.68 to –0.14, *P*<.01 and daughters: b=–0.32, 95% CI –0.59 to –0.04, *P*=.02; vaccine intentions, self: b=–4.20, 95% CI –7.53 to –0.87, *P*=.01 and daughters: b=–2.85, 95% CI –5.12 to –0.58, *P*=.01). Moreover, mothers’ intentions to vaccinate self may have increased when they considered the near-peer parents to be not credible (b=–0.50, 95% CI –0.99 to –0.01, *P*=.05).

The higher perceived credibility of the individual posts rated during the intervention also predicted increased social distancing by daughters (b=0.23, 95% CI 0.04-0.42, *P*=.02) but not mothers (b=0.07, 95% CI –0.09 to 0.23, *P*=.37). It also was associated with greater vaccine intentions for self (b=1.09, 95% CI 0.27-1.91, *P*=.01) but not for daughters (b=0.63, 95% CI –0.09 to 1.35, *P*=.09). However, there were no significant interactions between the credibility of posts and information sources for social distancing for self (credibility × government agency: b=–0.05, 95% CI –0.26 to 0.16, *P*=.62; credibility × near-peer parents: b=0.04, 95% CI –0.20 to 0.29, *P*=.72) and for daughters (credibility × government agency: b=–0.16, 95% CI –0.41 to 0.08, *P*=.19; credibility × near-peer parents: b=0.06, 95% CI –0.22 to 0.35, *P*=.65) or vaccine intentions for self (credibility × government agency: b=0.20, 95% CI –0.84 to 1.23, *P*=.71; credibility × near-peer parents: b=0.42, 95% CI –0.87 to 1.71, *P*=.52) and for daughters (credibility × government agency: b=0.15, 95% CI –0.79 to 1.09, *P*=.75; credibility × near-peer parents: b=–0.52, 95% CI –1.60 to 0.57, *P*=.35).

#### Effects of Engagement With COVID-19 Social Media Messages

Two measures of exposure to the social media posts, number of views of the posts, and number of reactions and comments to the posts were tested as moderators of the intervention’s effects on social distancing and vaccine intentions.

### Social Distancing

The number of views of posts by participants did not influence their reports of social distancing by self or daughters, but reports of social distancing by daughters was higher among mothers who had more reactions and comments (b=0.01, 95% CI 0.01-0.01, *P*=.04). There was no evidence that engagement moderated differences among information sources (*P*>.05).

### Vaccine Intentions

For views, the increase in vaccine intentions for self over time was attenuated when mothers viewed more posts across all conditions (b=–0.01, 95% CI –0.01 to –0.01, *P*=.01). This attenuation was stronger in the government agency group (self: b=–0.02, 95% CI –0.04 to 0.00, *P*<.001; daughters: b=–0.01, 95% CI –0.01 to –0.01, *P*=.01). By contrast, attenuation of the increase in vaccine intentions was less evident in mothers in the near-peer parents group who had more engagement (self: b=0.02, 95% CI 0.00-0.04, *P*<.01; daughters: b=0.02, 95% CI 0.00-0.04, *P*<.001). Engagement measured by reactions and comments did not affect changes in vaccine intentions (*P*>.05).

#### Moderation by Baseline Exposure to COVID-19 Media and Political Leaning

Potential moderation of change in social distancing and vaccine intentions by mothers’ general exposure to media reporting on COVID-19 and political leaning at baseline was also examined.

### Baseline COVID-19 Media Exposure

Baseline exposure to COVID-19 information in news media, averaged across 4 items, was similar across conditions on a 5-point scale (government agency mean 4.11, SD 0.88; near-peer parents mean 4.09, SD 0.91; news media mean 4.01, SD 0.82). Social distancing (self: b=0.46, 95% CI 0.36-0.56, *P*<.01; daughters: b=0.34, 95% CI 0.24-0.44, *P*<.01) and vaccine intentions (self: b=3.87, 95% CI 2.62-5.12, *P*<.001; daughters: b=2.80, 95% CI 1.93-3.66, *P*<.001) were higher at the 9-week posttest among mothers who reported more media exposure at baseline. However, baseline exposure did not affect differences by information source in either outcome.

### Political Leaning

Political leaning was normally distributed among mothers within each condition (government agency: conservative n=25, 25.3%, moderate n=54, 54.6%, liberal n=20, 20.2%; near-peer parents: conservative n=25, 25.8%, moderate n=48, 49.5%, liberal n=24, 24.7%; news media: conservative n=22, 22.2%, moderate n=46, 46.5%, liberal n=31, 31.3%). Mothers reported increased social distancing (self: b=0.40, 95% CI 0.28-0.52, *P*<.001; daughters: b=0.31, 95% CI 0.19-0.42, *P*<.001) and vaccine intentions (self: b=3.16, 95% CI 1.49-4.82, *P*<.001; daughters: b=2.37, 95% CI 1.21-3.53, *P*<.001) over baseline at the 9-week posttest when they expressed a more liberal than conservative political leaning. Political leaning moderated differences by information source for reports of social distancing by daughters. Mothers who were more liberal and assigned to the near-peer parents group reported greater social distancing by daughters at the final posttest (b=0.19, 95% CI 0.01-0.37, *P*=.04), while more liberal mothers in the government agency group reported reduced social distancing at the final posttest (b=–0.25, 95% CI –0.43 to –0.07, *P*<.01). Political leaning did not show any other effects on vaccine intentions for self (near-peer parents: b=0.13, 95% CI –0.18 to 0.44, *P*=.43; government agency: b=–0.11, 95% CI –0.42 to 0.20, *P*=.50) or daughters (near-peer parents: b=–0.03, 95% CI –0.30 to 0.24, *P*=.85; government agency: b=0.20, 95% CI –0.09 to 0.49, *P*=.18).

### Focus Group Results on Acceptability of the Social Media Messages

Of the 303 participants, 30 (9.9%) randomly selected participants (n=10, 33.3%, per treatment group) attended postintervention focus groups on reactions to the social media messages in the intervention. Coding of the 35 responses about what they liked most about the Facebook group (interrater reliability *κ*=0.82) revealed that the most common themes were a sense of community (n=15, 43%, responses) and program content or community manager (n=15, 43%, responses), followed by hearing opinions and perspectives that were different from the participants’ (n=5, 14%). Of the 30 responses on what the participants liked least about the Facebook group, the most frequent theme was that they did not dislike any aspect of the program (n=14, 47%), followed by hearing opinions that they disagreed with or feeling fearful of offending people who might disagree (n=8, 27%; *κ*=0.78). A small number of participants (n=5,17%) said they did not remember any content (n=3, 10%, responses were classified as “other”; eg, wished other moms engaged more). Finally, of 39 responses about what they learned in the Facebook group, the mothers more commonly mentioned facts about the vaccine (n=14, 36%), followed by general facts about COVID-19 (n=5, 13%), media literacy skills (n=5, 13%), and what other moms think about COVID-19 and vaccines (n=4, 10%; *κ*=0.87). A small number (n=5, 13%) said they had already heard all of the information in the messages, while a few (n=4, 10%) said they did not remember any of the content.

## Discussion

### Principal Findings

The results of this study must be interpreted within the context of the COVID-19 pandemic during the intervention. The relaxing of restrictions and ramping up of vaccination by March 2021 [22] may have made mothers feel that the risk from COVID-19 was diminishing, reflected in their lower perceived susceptibility to COVID-19 at 9 weeks. The EPPM asserts that health behavior is motivated by perceived risk [39,89], so this declining sense of susceptibility may have caused mothers and daughters to reduce their social distancing, a phenomenon seen in the H1N1 pandemic and other studies on COVID-19 [90-92]. Thus, these contextual factors may explain the failure to support our hypothesis of increased social distancing after the social media messages, which was seen in surveys [93,94]. By contrast, the expanding availability of the vaccine likely increased perceptions that mothers could get vaccinated, which produced greater self-efficacy for vaccination over time. This may have motivated stronger intentions to get vaccinated during the study. However, increased intentions appeared to occur mostly among mothers who had moderate-to-high intentions at baseline, while mothers with initially low intentions became more resistant over time.

The information source linked to the social media messages in the Facebook posts did not have a clear effect on mothers. Government sources may have attenuated the decline in social distancing mothers reported for daughters, while near-peer parents possibly amplified the decline. The government sources selected for the social media messages advocated for social distancing and thus rebutted local government decisions to relax restrictions. In a previous study, attention to government sources improved social distancing behaviors [50]. However, the near-peer parents may have increased participants’ decisions to abandon social distancing, despite presenting messages supporting social distancing. It may be that other parents in the mothers’ lives were strongly opposed to social distancing and hearing from “parents” in the social media posts made several mothers more aware of the parents’ general opposition. By contrast, mothers with initially high intentions to get themselves vaccinated had weaker intentions at the end of the intervention period when receiving information from government sources. Their intentions could have declined because many of these mothers were vaccinated during the study, making intentions less relevant. Other studies have found that social media and online sources have limited impacts on perceptions related to COVID-19 prevention and sometimes result in lower knowledge [45,48,51]. Past research showed that in the United States, news media preferences affected COVID-19 knowledge and altered COVID-19 prevention behaviors, when comparing conservative news media outlets with outlets with more moderate or liberal political views [95,96]. We attempted to control these varying preferences by using randomization and linking to news media with different political perspectives from moderately liberal to moderately conservative. However, the heterogeneity of perceptions may have made it difficult to discern a consistent effect in the news media condition.

The intervention’s social media messages seemed to affect mothers when they contained information sources that mothers considered credible, regardless of which source they received. Similarly, a recent study found that trust in specific sources of information on the pandemic results in higher COVID-19 health literacy [49]. Past research showed that risk communication must build trust in the government, medical organizations, and science to improve adherence to protection measures [97-99]. Consistent with the EPPM [39], information from high-credibility sources may make it more difficult to engage in fear control to reduce perceived severity, which increased during the intervention, through source derogation and dismissal. Instead, it may have motivated mothers to take steps to control the danger through social distancing and vaccinations, especially when perceived response costs declined.

The findings of this trial suggest that when using social media to improve COVID-19 prevention behaviors and vaccine uptake, campaign planners should, as a general strategy, select sources that recipients feel are trustworthy and accurate and construct messages that maintain these perceptions of high credibility. The sense of community cited by several mothers in follow-up interviews as something they liked about the private groups might have contributed to credibility, because goodwill toward others has been a dimension of source credibility [100]. In addition, mothers who liked the ability to hear perspectives different from their own may have seen the groups as a safe place to experience differing opinions, again expressing this sense of goodwill. Some mothers were hesitant to offend people who might disagree with their opinions, implying there may have been a norm of civility in the private groups that contributed to credibility as well. However, campaign planners need to avoid information overload, which has been associated with consuming certain sources, and a larger number of sources, which can cause recipients to actively avoid information [45,47,101-103].

The general conclusion that highly credible sources are most effective, however, may not always hold when considering near-peer parents as sources of information about COVID-19 (ie, parents in this case). In this study, mothers who felt near-peer parents were not credible initially may have been more influenced by the social media messages. It may be that mothers who generally considered near-peer parents to be less credible on COVID-19 may have found the near-peer parents included in the experimental posts to be more believable than they expected. Prior research has shown that individuals who argue for a position that they are not expected to hold are more influential, especially when the arguments are high quality [104]. In addition, a positive violation of expectations in persuasive messages can make individuals appear more credible and hence persuasive [105-108].

The finding that regardless of the information source, mothers’ engagement with the social media messages in the Facebook private groups was associated with an attenuated reduction in social distancing was consistent with other studies in which engagement improved social media’s and other digital interventions’ effectiveness [109-113]. However, engagement effects in this trial may have been limited by the generally high degree of exposure mothers had in all groups. Engaging with a social media intervention may be different from engaging with other forms of digital interventions, such as websites or online training. Reactions and comments are considered more involved engagement than just viewing posts, as the former represents conversation that may be more intrinsically engaging, while the latter is merely information consumption [114]. Viewing was more common than reacting and commenting in this study, and the 2 forms of engagement may have different motivations. Views may reflect information needs, while reaction or comments may fulfill social needs [115-117]. It is important to note that views, reactions, and comments are behavioral measures of online engagement, but researchers have recently argued that engagement is multidimensional and involves emotional and cognitive experiential processes that are better captured with self-reporting and other measures [118-120]. For example, mothers may have viewed a post and then discussed it with friends or family. Simply frequent, sustained online behavioral engagement may not capture the complex nature of engagement. There is a need to identify what constitutes effective engagement with social media [119,121].

Finally, 2 other contextual trends were apparent in the study results. Mothers who had paid more attention to COVID-19 information in the media prior to the study had higher social distancing and vaccine intentions by the final posttest. It may be that greater attention to the COVID-19 information environment provided mothers with more information that promoted COVID-19 prevention, including vaccine intentions. A recent study found that individuals with high perceived COVID-19 risk and greater prevention behaviors reported consuming information on COVID-19 from multiple sources [60]. Finally, there is ample evidence, including in this trial, that conservative political leaning is a major barrier to COVID-19 prevention [122,123]. This appears to be a robust tendency unaffected by different information sources.

### Limitations and Strengths

The trial had some limitations. The design lacked a control group that did not receive messages on COVID-19, which made it challenging to determine whether the messages affected social distancing and vaccine intentions irrespective of the information source. The short duration of the intervention may have achieved only small effects. Although the sample was moderate in size and from a number of US states, generalizability was limited by enrolling mothers of teen daughters who may have been more attentive to the social media messages because they had elevated concerns about COVID-19 risks for their families. Whether individuals who are not parents would be affected in the same way is unknown. Mothers had already participated in a trial on other adolescent health topics, so the sample may have been biased to mothers with high interest in adolescent health. Most mothers were originally recruited from the Qualtrics survey panel, which tends to have a relatively high socioeconomic status, and nearly all mothers were non-Hispanic White because of the original trial’s focus on indoor tanning. Although we varied the source of information contained in the posts, all posts were delivered through the Facebook platform, making it the primary source of the intervention and possibly undermining the experimental comparison. The multiple posttest measures may have introduced a testing effect (ie, reactivity) that increased the mothers’ attention to the experimental messages because they knew they would be assessed every 3 weeks. All assessments were self-reporting, although many outcomes were intrapsychic processes (eg, perceptions, opinions, and intentions) measurable only through reports from mothers. We did use published scales, when available.

These limitations were offset somewhat by strengths of the study. Mothers were enrolled and pretested prior to the intervention, allowing for prospective tests of social media’s effects, and were randomly assigned to 3 prominent sources of pandemic information, which improved the validity of these comparisons. A mixed methods approach was used to understand the impact of the social media messages on mothers. Finally, multiple posttests provided information on changes produced by the intervention over time.

### Conclusion

There were several lessons learned to inform future trials using social media interventions. The group size of approximately 100 mothers was sufficient to achieve high viewership and active participation by group members over 9 weeks, although, as noted, the COVID-19 topic may have been generally interesting to them. Future studies should test how long engagement with a social media intervention can be sustained. In our parent trial with messages on general adolescent health topics, engagement declined over the first 6 months [70]. Participants were willing to remain in the group once they joined it, increasing the likelihood that the social media messages reached and affected them. Many large social media feeds are curated, and it required substantial time to manage the experimental Facebook groups, at least 10 hours a week by the community manager. The community manager played an important role in engaging participants by personalizing the experimental messages by highlighting that she was a mother and showing her picture.

Social media has been a source of information and misinformation even before the COVID-19 pandemic, but concerns over its role in the pandemic have been elevated as millions of Americans have been exposed to deceptive information, which some people can find believable [24,31,76,124,125]. Social media can affect vaccine-related decisions [126-128], and experts and researchers have called for efforts to correct information on social media [25,32,33,129]. In this context, the trial showed that a series of social media messages can be used to support pandemic responses when posts are based on health behavior change theories and information sources are tailored to the audiences’ existing credibility beliefs.

## Appendix

Multimedia Appendix 1 Measurement scales.

## Abbreviations

CDC: Centers for Disease Control and Prevention
CONSORT: Consolidated Standards of Reporting Trials
EPPM: Extended Parallel Process Model
IT: indoor tanning
NPI: nonpharmaceutical intervention
SCT: social cognitive theory
