# Supplementary material for: Promoting Social Distancing and COVID-19 Vaccine Intentions to Mothers: Randomized Comparison of Information Sources in Social Media Messages
Source: JMIR Infodemiology. 2022 Aug 23;2(2):e36210. doi: 10.2196/36210 (PMC9400429; doi:10.2196/36210)
Supplement: Multimedia Appendix 1 [file infodemiology_v2i2e36210_app1.docx]

**Multimedia** **Appendix 1: Measurement scales.**

**COVID-19 Prevention Behaviors for Self (never=1, always=5)**

In the past 3 weeks, since the middle of January, how frequently have you taken the following actions?

- Studied or worked remotely from home
- Deliberately canceled or postponed a social event
- Avoided places where people gather
- Kept at least 6 feet away from other people
- Ate indoors at a restaurant
- Attended a social event indoors with 10 or more people who do not live in my house
- Wore a mask that covers my face and nose when out in public
- Washed my hands more often than usual

**COVID-19 Prevention Behaviors for Daughter (never=1, always=5)**

To the best of your knowledge, in the past 3 weeks, since the middle of January, how frequently has your daughter taken the following actions?

- Studied or worked remotely from home
- Deliberately canceled or postponed a social event
- Avoided places where people gather
- Kept at least 6 feet away from other people
- Ate indoors at a restaurant
- Attended a social event indoors with 10 or more people who do not live in my house
- Wore a mask that covers her face and nose when out in public
- Washed her hands more often than usual

**Vaccine Antecedents (1=strongly disagree, 5=strongly agree)**

- I am completely confident that vaccines are safe.
- My immune system is so strong, it also protects me against diseases.
- Everyday stress prevents me from getting vaccinated.
- When I think about getting vaccinated, I weigh benefits and risks to make the best decision possible.
- When most other people are vaccinated, I don’t have to get vaccinated, too.
- I get vaccinated because I can also protect people with a weaker immune system.
- Vaccination is a collective action to prevent the spread of diseases.

**Intention to Vaccinate (0=definitely would not get vaccine, 100=definitely would get the vaccine)**

If a vaccine for COVID-19 were available to you today, what is the likelihood that you would get vaccinated?

If a vaccine for COVID-19 were available to your daughter today, what is the likelihood that your daughter would get vaccinated?

**Family Communication About COVID-19 NPIs and Vaccination (0=no, 1=yes)**

In the past 3 weeks, that is from the middle of January, did you talk with your daughter about any of the following topics?

- Practicing social distancing, handwashing, and mask wearing and getting vaccinated for COVID-19 can help people avoid getting COVID-19.
- Practicing social distancing, handwashing, and mask wearing and getting vaccinated for COVID-19 are simple.
- Practicing social distancing, handwashing, and mask wearing and getting vaccinated for COVID-19 are low cost.
- Checking the accuracy of information about COVID-19 that they see online and in the media is important.
- Most people in our community are practicing social distancing, handwashing, and mask wearing to prevent getting infected with COVID-19.
- COVID-19 vaccines will be safe and effective.
- Many people in our community will get a COVID-19 vaccine when it is available.

**Perceived Risk for COVID-19 Infection (1=strongly disagree, 5=strongly agree)**

Perceived Severity

- COVID-19 has severe consequences.
- COVID-19 is harmful.

Perceived Susceptibility

- I am likely to get COVID-19.
- The chances of getting COVID-19 are high.

**Self-Efficacy for NPIs (1=strongly disagree, 5=strongly agree)**

- I would be able to take actions to reduce the risk of catching COVID-19 if I wanted to.
- Taking action to reduce the risk of catching COVID-19 would be difficult for me.

**Self-Efficacy for COVID-19 Vaccination for Self (1=strongly disagree, 5=strongly agree)**

- I would be able to get a vaccine for COVID-19 if the vaccine was available and I wanted to.
- Getting vaccinated for COVID-19 would be difficult for me, if the vaccine was available.

**Self-Efficacy for COVID-19 Vaccination for Daughter (1=strongly disagree, 5=strongly agree)**

- I would be able to get my daughter vaccinated for COVID-19 if the vaccine was available and I wanted to.
- Getting my daughter vaccinated for COVID-19 would be difficult for me, if the vaccine was available.

**Response Efficacy of COVID-19 NPIs (1=strongly disagree, 5=strongly agree)**

- Taking actions to avoid infection reduces my chances of catching COVID-19.
- Taking actions to avoid COVID-19 infection protects myself and others around me.

**Response Cost of COVID-19 NPIs (1=strongly disagree, 5=strongly agree)**

- The benefits of taking actions to avoid COVID-19 infection outweigh the costs.
- I don’t take actions to avoid COVID-19 infection, because they would impact my work.
- I don’t take actions to avoid COVID-19 infection, because they are against my political beliefs.

**Source Credibility of the Government, Near-Peer Parents, and News Media for COVID-19 Information (1-5 scale)**

Please indicate how you feel about the information about COVID-19 that you received from the government/parents like you/news media.

- Not Trustworthy – Trustworthy
- Inaccurate – Accurate
- Biased – Not Biased

**Exposure to COVID-19 Messages in the Media (1=do not pay any attention at all, 5=pay a lot of attention)**

Please tell us how much attention you pay to each the following information types in the media.

- The number of infected individuals or cases of COVID-19
- Patients with COVID-19
- Doctors and nurses treating COVID-19
- Government officials working on COVID-19
- Vaccines against COVID-19
- Actions people can take to prevent becoming infected with COVID-19

**COVID-19 Information Overload (1=strongly disagree, 5=strongly agree)**

- I find that I am overwhelmed by the amount of information about COVID-19 that I process on a daily basis from many different channels and sources.
- I receive too much information regarding the COVID-19 pandemic to form a clear picture of what is happening.

**COVID-19 Information Excessiveness (1=strongly disagree, 5 = strongly agree)**

- It has gotten to the point where I don’t even care to hear new information about COVID-19.
- There is not enough time to do all of the things recommended to prevent COVID-19.
